# Supplementary material for: Low intensity repetitive transcranial magnetic stimulation modulates brain-wide functional connectivity to promote anti-correlated c-Fos expression
Source: Sci Rep. 2022 Nov 29;12:20571. doi: 10.1038/s41598-022-24934-8 (PMC9708643; doi:10.1038/s41598-022-24934-8)
Supplement: Supplementary file 3 — Supplementary Figure 1. [file 41598_2022_24934_MOESM3_ESM.docx]

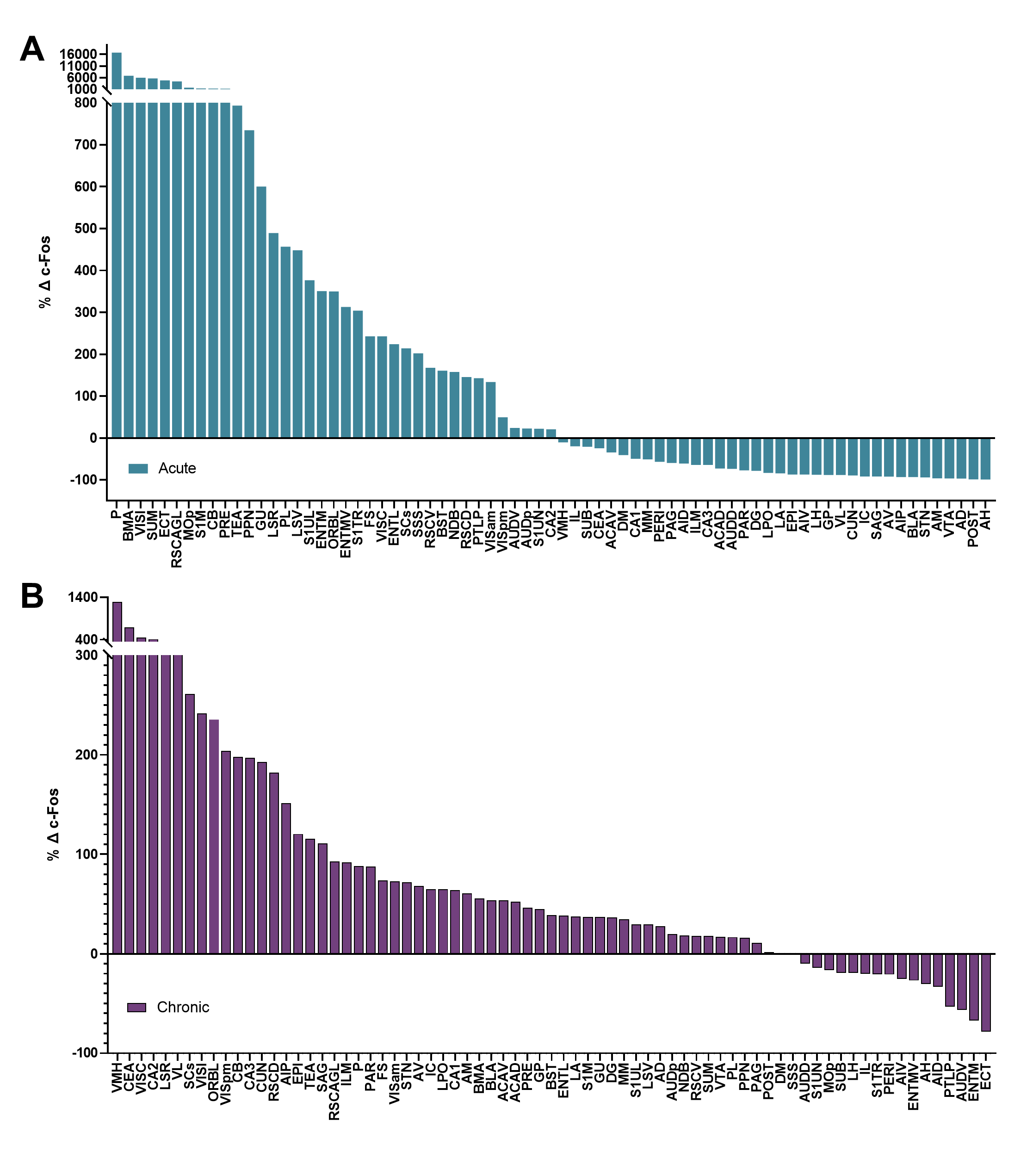


**S1 Fig. Mean percentage difference between active and sham LI-rTMS for (A) acute and (B) chronic groups for all analysed brain regions.** Regions are organised by magnitude of percentage change. Note acute and chronic groups have different y-axis scales.
