# Supplementary material for: Low intensity repetitive transcranial magnetic stimulation modulates brain-wide functional connectivity to promote anti-correlated c-Fos expression
Source: Sci Rep. 2022 Nov 29;12:20571. doi: 10.1038/s41598-022-24934-8 (PMC9708643; doi:10.1038/s41598-022-24934-8)
Supplement: Supplementary file 5 — Supplementary Figure 2. [file 41598_2022_24934_MOESM5_ESM.docx]

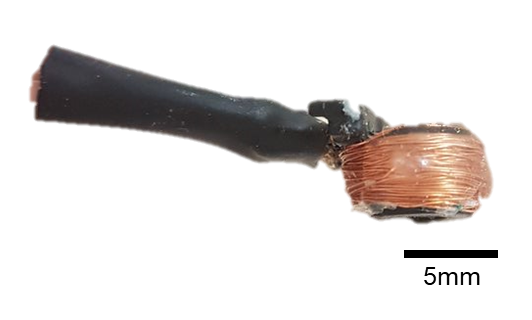


**S2 Fig.** **Image depicting custom low-intensity repetitive transcranial magnetic stimulation (LI-rTMS) coil.** Scale bar = 5mm. 300 copper windings, external diameter, 8 mm; internal diameter 5 mm.
